# Complete Genome Sequence of the Type Strain of *Macrococcus canis*

**DOI:** 10.1128/genomeA.01507-17

**Published:** 2018-01-18

**Authors:** Stefanie Gobeli Brawand, Lorenz Rychener, Sybille Schwendener, Roman Pantůček, Vincent Perreten

**Affiliations:** a Institute of Veterinary Bacteriology, Vetsuisse Faculty, University of Bern, Bern, Switzerland; b Department of Experimental Biology, Faculty of Science, Masaryk University, Brno, Czech Republic

## Abstract

The first complete genome sequence of the recently described *Macrococcus canis* species has been determined for the strain KM45013^T^ (=DSM 101690^T^ = CCOS 969^T^ = CCUG 68920^T ^= CCM 8748^T^). The strain was isolated from a dog with rhinitis and contains a putative γ-hemolysin and a *mecB*-carrying staphylococcal cassette chromosome *mec* element (SCC*mec*_KM45013_).

## GENOME ANNOUNCEMENT

*Macrococcus canis* is the latest member described in the *Macrococcus* genus, which now contains a total of eight species (*M. caseolyticus*, *M. carouselicus*, *M. equipercicus*, *M. bovicus*, *M. brunensis*, *M. hajekii*, *M. lamae*, and *M. canis* (1). *M. canis* has been found on the skin of healthy dogs and has also been associated with several types of skin- and mucosa-related infections (2). To our knowledge, it is the only *Macrococcus* species that produces hemolysis that is visible on sheep blood agar plates (1). *M. canis* also has the ability to acquire antibiotic resistance genes, including the methicillin resistance gene *mecB*. *mecB* has been shown to be present on the staphylococcal cassette chromosome *mec* element (SCC*mec*) in strain KM45013 (SCC*mec*_KM45013_) (3). At the time of isolation, strain KM45013 was identified as *M. caseolyticus* and was recognized as a new *Macrococcus* species in early 2017. Strain KM45013 is now the type strain of *M. canis* and was assigned the name KM45013^T^ (= DSM 101690^T^ = CCOS 969^T^ = CCUG 68920^T^ = CCM 8748^T^) (1). We report here the complete genome sequence of *M. canis*.

PacBio raw reads (80,881 reads) (Pacific Biosciences, USA) were assembled using Canu version 1.4 (4). After circularization, Ion Torrent reads (3,351,349 reads) (Thermo Fisher, USA) were mapped against the contig from the PacBio assembly using Bowtie 2 version 2.2.4 (5). To spot possible frameshifts, single nucleotide variants were called using SAMtools version 1.3 (6), and the errors were corrected manually with the visual aid of Geneious version 10.1.2 (7). The resulting full genome is 2,363,414 bp, with a GC content of 37.1% and a coverage of >350-fold. No plasmids were detected. The Prokka pipeline version 1.11 (8), using the previously published SCC*mec*_KM45013_ of the strain KM45013 (GenBank accession no. HG970732), and UniProtKB as an additional database were used to annotate the corrected sequence. A total of 2,438 coding sequences and 120 RNAs, including 59 tRNAs, were predicted.

In addition to the methicillin resistance gene *mecB* conferring resistance to β-lactam antibiotics (3), the genome of *M. canis* strain KM45013^T^ contains genes for a bicomponent γ-hemolysin (*hlgB* at complement positions 98969 to 99922 and *hlgC* at complement positions 99935 to 100879 in GenBank accession no. CP021059) explaining the hemolytic phenotype observed on sheep blood agar plates. Two putative prophages were also predicted using the PHASTER tool (9) within the genome at positions 1320695 to 1364425 and 1513641 to 1551467 in GenBank accession no. CP021059.

The complete genome of strain KM45013^T^ serves as a baseline for future molecular and epidemiological studies using comparative genomic analysis of *M. canis*, and it permits increased knowledge on this novel opportunistic animal pathogen.

### Accession number(s).

The whole-genome sequence of *M. canis* strain KM45013^T^ has been deposited in DDBJ/ENA/GenBank under the accession no. CP021059.
